# Delays in Blood Work and Disease Burden: A Cross-Sectional Analysis of Unmet Blood Work Need and Seven Key Health Conditions Across 21 Countries

**DOI:** 10.3389/ijph.2024.1607667

**Published:** 2025-01-06

**Authors:** Eunice Chung, Louisa Ewald, Nicholas J. Kassembaum, Taylor Noyes, Emmanuela Gakidou, Ali H. Mokdad

**Affiliations:** ^1^ Institute for Health Metrics and Evaluation, University of Washington, Seattle, WA, United States; ^2^ Department of Health Metrics, University of Washington, Seattle, WA, United States

**Keywords:** blood work, health disparities, global burden of disease, delays in care, hemoglobinopathies

## Abstract

**Objectives:**

This study analyzes survey data across 21 countries to explore correlations between delays in blood testing and the prevalence of seven health conditions: thalassaemias, sickle cell disorders, malaria, HIV, high fasting plasma glucose, impaired kidney function, and high LDL cholesterol.

**Methods:**

We analyzed Pandemic Recovery Survey data via multivariable logistic regression to compare blood test delays between individuals with and without medical conditions, while adjusting for sociodemographic factors. We also examined the disease burden using disability-adjusted life years (DALYs) and summary exposure values (SEV) rates.

**Results:**

Our findings indicate profound disparities, with over 60% of respondents in Egypt, Nigeria, and India reporting they have never undergone blood tests. Individuals with existing medical conditions are significantly more likely to experience delays in blood work.

**Conclusion:**

There is a pronounced gap in blood work accessibility, particularly in countries with high disease burdens. Findings suggest an urgent need for interventions to improve routine blood test access for high-risk populations to reduce the underdiagnosis of significant medical conditions. Prioritizing timely and accessible blood testing can serve as a step towards mitigating healthcare disparities.

## Introduction

Blood testing is a foundational aspect of both routine and preventive care, enabling the screening for medical conditions and the monitoring of chronic disease progression. The timeliness of blood work can impact various clinical outcomes, including the duration of hospital stay, mortality rates, the intensity of care required, and the quality of communication between physicians and patients [1, 2]. However, barriers to accessing blood work can notably delay the timely diagnosis and treatment of medical conditions [3–5]. This issue is particularly pronounced in diseases such as malaria or dengue fever, where the speed of diagnosis directly impacts the management and treatment outcomes.

Thalassaemias and sickle cell disorders, two of the world’s most prevalent blood diseases, can lead to severe health consequences if there is a delay in blood work [6, 7]. Countries with high Disability Adjusted Life Years (DALY) rates from thalassaemias and sickle cell disorders are particularly susceptible to high financial burden and costs in treatment, exacerbated by delays in blood work [8, 9]. Malaria and HIV are also diseases that require timely and consistent monitoring through blood tests to manage potential infections effectively [10, 11]. Moreover, blood work is essential for diagnosing high fasting plasma glucose, high LDL cholesterol, and impaired kidney function.

Before the COVID-19 pandemic, there were gaps in diagnostic testing globally, with the availability of medical diagnostics closely correlated with a country’s income level in primary care settings [12]. The pandemic has further disrupted essential and preventive healthcare services worldwide, delaying diagnostic testing for many individuals [13]. Despite the recognized importance of timely blood work, there is a lack of comprehensive studies addressing the unmet need for blood work following the COVID-19 pandemic [14].

Previous studies on the accessibility of blood work have been limited in scope, mainly focused on the surveillance of blood diseases, patient experiences, workplace wellbeing, or the monitoring of specific diseases [15–17]. [10] To our knowledge, there are no studies that examine the relationship between access to blood work and the burden of diseases and conditions reliant on blood tests for diagnosis – such as high fasting plasma glucose (FPG), impaired kidney function, high LDL cholesterol, malaria, HIV, sickle cell disorders, and thalassaemias. Comparing the rates of blood work delays with the global DALYs and summary exposure values (SEVs) of these diseases and conditions emphasizes the essential role of early detection and diagnosis through blood tests.

This study analyses data from the Pandemic Recovery Survey, an online survey conducted across 21 countries, to identify determinants of unmet blood work needs. We aim to compare these findings with the global disease burden of high fasting plasma glucose (FPG), impaired kidney function, high LDL cholesterol, malaria, HIV, sickle cell disorders, and thalassaemias from the Global Burden of Disease 2021 study [18]. By comparing the self-reported data from the Pandemic Recovery Survey with the DALYs of the aforementioned medical conditions, we provide needed context on the relationship between access to blood work and the corresponding disease burden faced in those areas.

In evaluating the delays in blood work, our study includes a wide array of diseases, including highly infectious diseases such as HIV, vector-borne diseases, and significant non-communicable conditions like thalassaemias and sickle cell disorders. This selection reflects the diverse global health challenges these diseases represent and underscores the critical role of blood testing in their management. Recognizing the varied approaches to blood testing across nations-where specific testing systems and periods may differ-our study aims to shed light on the universal importance of timely blood work across these diverse health contexts. This analysis underscores the urgent need for interventions and policies to ensure routine access to blood testing for high-risk populations, as a step toward addressing healthcare disparities and improving the diagnosis of significant medical conditions.

## Methods

### Study Design and Participants

This cross-sectional, internet-based survey was conducted as part of the Pandemic Recovery Survey (PRS) across 21 countries from March to May 2023 [19]. The PRS sought to capture the population-level impact of the COVID-19 pandemic, focusing on economic, educational, and health outcomes. The target population included active Facebook users aged 18 and over in the selected 21 countries. Participants were identified through the Facebook Active User Base (FAUB) which was divided into strata by location and gender to help ensure a balanced coverage of genders in the final sample. Random samples were drawn from each gender strata and participants received an invitation via notification on their Facebook account. Participants were not able to take the survey twice or send the link to others. Participants were only excluded from the study if they were under the age of 18 or did not consent to participate in the study.

The questionnaire was translated and checked by native speakers in 15 languages and data were collected through an online survey platform, Qualtrics. Researchers did not have access to any identifying information, including the participant’s Facebook information. Meta did not have access to any data. All participants provided informed consent prior to taking the survey. The questionnaire was pilot tested with a percentage of the total sample in all countries before launch.

The 21 countries were chosen based on population, geographic distribution, number of active Facebook users, and estimated healthcare disruption due to the COVID-19 pandemic. The study design and questionnaire were submitted and approved by the Institutional Review Board of the University of Washington (STUDY00016693).

### Measures

#### Delayed Blood Work

Questions regarding health service use were asked to respondents who reported an existing medical condition (heart attack, stroke, high blood pressure, cancer, diabetes, lung disease, dementia or Alzheimer’s disease, mental health condition, addiction or substance use disorder, liver disease, and kidney disease). All respondents were also asked about preventive healthcare services, including blood work, that they had received in their lifetime. The term “blood work” encompasses a range of diagnostic tests. Particular parameters, such as Complete Blood Count (CBC), was not specified due to the study’s broad, cross-country scope and the variability of testing practices across regions. Delayed care is defined as a respondent indicating that they needed a service in the previous 6 months but could not receive it for various reasons. Never receiving blood work is defined as a respondent answering that they have never received blood work in their life.

Demographic questions included age (18–24 years, 25–29 years, 30–49 years, and 50 years and older), gender (male, female), educational attainment (primary or less, secondary, college or more), financial stability (easy to afford household expenses, somewhat difficult, or very difficult), and food security (enough to eat, sometimes enough to eat, or not enough to eat over the preceding 30 days).

### Statistical Analyses

#### Determinants of Never Receiving Blood Work

A multivariable logistic regression was conducted to assess the association between sociodemographic factors and the likelihood of ever having blood work. Variables in the analysis included gender, age, education level, financial security, whether the respondent has a current medical condition, and whether the respondent experienced delayed blood work.

We conducted the multilevel logistic regression using the glmer function from the lme4 package, with individuals nested within countries. Random effects were summarised with variance components, and country-specific random effects were reported relative to the global intercept. We analysed the data using R software and the survey package [20, 21].

#### Burden of Diseases Where Blood Testing Is Routinely Indicated

We used the results from GBD 2021 between 1990 and 2021 to evaluate the burden in terms of age-standardised DALYs rate of four types of diseases—thalassaemias, sickle cell disorders, malaria, HIV—and age-standardised SEV rates of three types of risk factors—impaired kidney function, high fasting plasma glucose, and high LDL cholesterol—where blood work is critical for diagnosis or subsequent treatment. Our study design specifically included a range of diseases to comprehensively assess the impact of blood work delays on disease burden globally. The selection was based on the prevalence of these conditions across the 21 countries surveyed and their reliance on blood testing for diagnosis and monitoring. This approach allowed us to capture the diverse testing protocols and periods employed across different healthcare systems, particularly in addressing diseases endemic to certain regions.

The estimates for both DALYs and SEVs were produced by sex (i.e., male and female) and age in 204 countries and territories. Final estimates were produced as age-standardised rates per 100,000 population. We assessed the burden of blood diseases through age-standardised DALY rates and associated risk factors through age-standardised SEV rates. One DALY represents the loss of the equivalent of 1 year of full health. DALYs for a disease or health condition are the sum of the years of life lost due to premature mortality (YLLs) and the years lived with a disability (YLDs) due to prevalent cases of the disease or health condition in a population [22]. SEV represents the average level of exposure to a particular risk factor across a specific population, weighted by the severity of that risk factor’s contributing to disease or health conditions. It provides a summary measure of how extensively a population is exposed to a risk factor, taking into account both the prevalence and the severity (or risk) associated with that exposure.

#### Association Between Blood Work Rates and Burden of Diseases

For each disease or disorder included in the analysis—thalassaemias, sickle cell disorders, malaria, HIV, impaired kidney function, high fasting plasma glucose, and high LDL cholesterol—we categorised the country-level burden into into tertiles (low, mid, or high) based on the DALY or SEV values. Compared to the DALY or SEV value for all countries, the third with the lowest values were assigned “low burden,” those with the next third were assigned “mid burden,” and those with the highest third of values were assigned “high burden.” Minimum, maximum, and cutoff values for each disease burden category can be found in Supplementary Appendix Table S1.

### Data Sources

The GBD conducts systematic reviews and opportunistic searches, and incorporates data shared by collaborators and WHO. The DALYs rate per 100,000 people reflects the iterations of data seeking effort through GBD 2021, where more details have been published [18]. Thalassaemias, sickle cell disorders, malaria, and HIV are classified as causes in the GBD framework, and their DALYs rates are reported in GBD 2021 [18]. High fasting plasma glucose (FPG), impaired kidney function, and high LDL cholesterol are reported as summary exposure values (SEVs) in the GBD framework because they are identified as risk factors.

### YLLs, YLDs, and DALYs

The methods used to calculate years of life lost (YLLs), years lived with disability (YLDs), and disability-adjusted life-years (DALYs) are detailed elsewhere [18, 22]. YLLs were calculated by multiplying the number of deaths by the standard life expectancy at the age at which death occurs. YLDs were determined by multiplying the prevalence of each specific outcome of the disease (sequela) by its associated disability weight. YLD calculations were adjusted for coexisting health conditions, assuming that these conditions are independent and combining them using a multiplicative approach. The total DALYs were calculated by adding together the YLLs and YLDs. The DALYs rate is expressed as the number of DALYs per 100,000 individuals. This standardisation allows for comparisons of disease burden across different populations and geographical regions by providing a measure of the total health loss per 100,000 people due to the medical conditions of interest.

### Summary Exposure Values (SEVs)

The SEV is expressed as a percentage, ranging from 0% to 100%, where 0% indicates no exposure in the population, and 100% represents maximum exposure or risk. The unit for the SEVs rate is expressed as the level of SEVs per 100,000 individuals. The standardisation allows for comparison of the risk factors across different populations and geographical regions by providing a measure of the likelihood of being exposed to the target risk per 100,000 people.

## Results

### Overview—Unmet Blood Work Needs as an Indicator for Delays in Diagnosis Through Blood Testing

The current study analyses data from a total of 295,485 people across 21 countries, as detailed in Table 1. Over half of respondents from Egypt, Nigeria, the Philippines, South Africa, India, and the UK have never received blood work. Egypt has a staggering rate of 80% of respondents never having had blood work done, closely followed by Nigeria (77%), the Philippines (75.2%), South Africa (73.8%), and India (73.7%).

**TABLE 1:** Sample size and proportion of never receiving blood work across 21 countries (2023).

Country name	Sample size	Percentage never received blood work (95% confidence interval)
Argentina	10,365	8.47% (8.06, 8.88)
Brazil	17,823	9.55% (8.8, 10.3)
Chile	9,813	17.81% (16.38, 19.24)
Colombia	14,701	20.89% (19.08, 22.7)
Egypt	16,855	80.49% (71.45, 89.53)
Germany	7,836	13.17% (12.51, 13.83)
India	21,629	74.00% (67.4, 80.6)
Indonesia	21,106	31.47% (27.63, 35.31)
Italy	14,509	6.93% (5.59, 8.27)
Japan	8,843	15.17% (14.91, 15.43)
Mexico	18,723	21.75% (18.93, 24.57)
Nigeria	16,734	77.73% (68.55, 86.91)
Peru	13,161	18.82% (16.95, 20.69)
Philippines	25,308	75.03% (68.15, 81.91)
Poland	12,809	6.75% (6.0, 7.5)
South Africa	17,907	73.69% (65.54, 81.84)
Spain	7,555	4.65% (4.28, 5.02)
Türkiye	9,317	22.31% (19.92, 24.7)
United Kingdom	6,622	50.99% (40.62, 61.36)
United States	8,736	17.09% (15.84, 18.34)
Viet Nam	15,133	30.50% (27.7, 33.3)

Table 1 shows the sample size and the proportion of respondents indicating that they have never received blood work. Data are from the Pandemic Recovery Survey, 21 countries, 2023.

**FIGURE 1:**
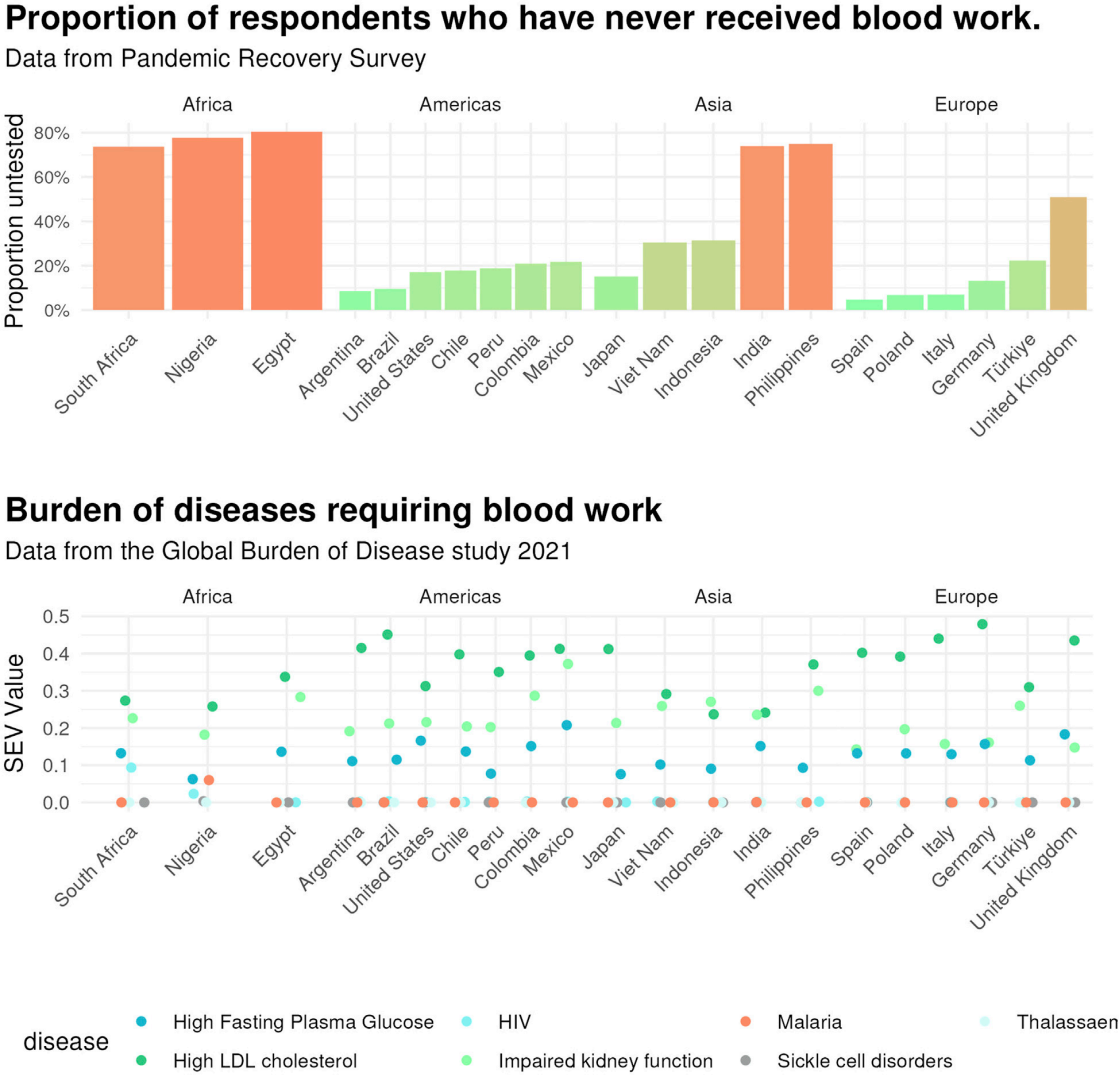
Proportion of respondents never receiving blood work and disease burden by country. *Upper.* Age-standardised proportion of respondents who have never received blood work by country. Data are from the Pandemic Recovery Survey across 21 countries, 2023. *Lower.* Proportion of respondents never receiving blood work by disease in terms of age-standardised disability-adjusted life years (DALY) rates (per 100,000) by location, both sexes combined for thalassaemias, sickle cell disorders, malaria, and HIV, and age-standardised summary exposure values (SEV) rates (per 100,000) by location, both sexes combined for high low-density lipoprotein (LDL) cholesterol, high fasting plasma glucose, and impaired kidney function. Data are from the Global Burden of Disease 2021 study.

We then compared the pattern of participants receiving blood work with seven types of conditions (thalassaemias, sickle cell disorders, malaria, HIV, high fasting plasma glucose, impaired kidney function, and high LDL cholesterol), as shown in Figure 1. South Africa, Nigeria and the Philippines are highly burdened by HIV, while the UK is highly burdened by high LDL cholesterol. The other two countries with staggering rates of absent blood work are India and Egypt; they both are highly burdened by high fasting plasma glucose (FPG) and sickle cell disorders. Malaria carries a disproportionately high share of disease burden in Nigeria, whereas the Philippines and Egypt continue to have a heavy burden due to impaired kidney function. South Africa, the Philippines, Nigeria, and Egypt have a high burden of thalassaemia.

### Access to Blood Work

The PRS indicates that a significant portion of the global population may be underserved in terms of essential blood work, with approximately 45.4% of respondents reporting they have never undergone any form of blood testing. South Africa, India, Nigeria, the Philippines, and Egypt have over 60% of their age-standardised population who have never received any blood work before.

**FIGURE 2:**
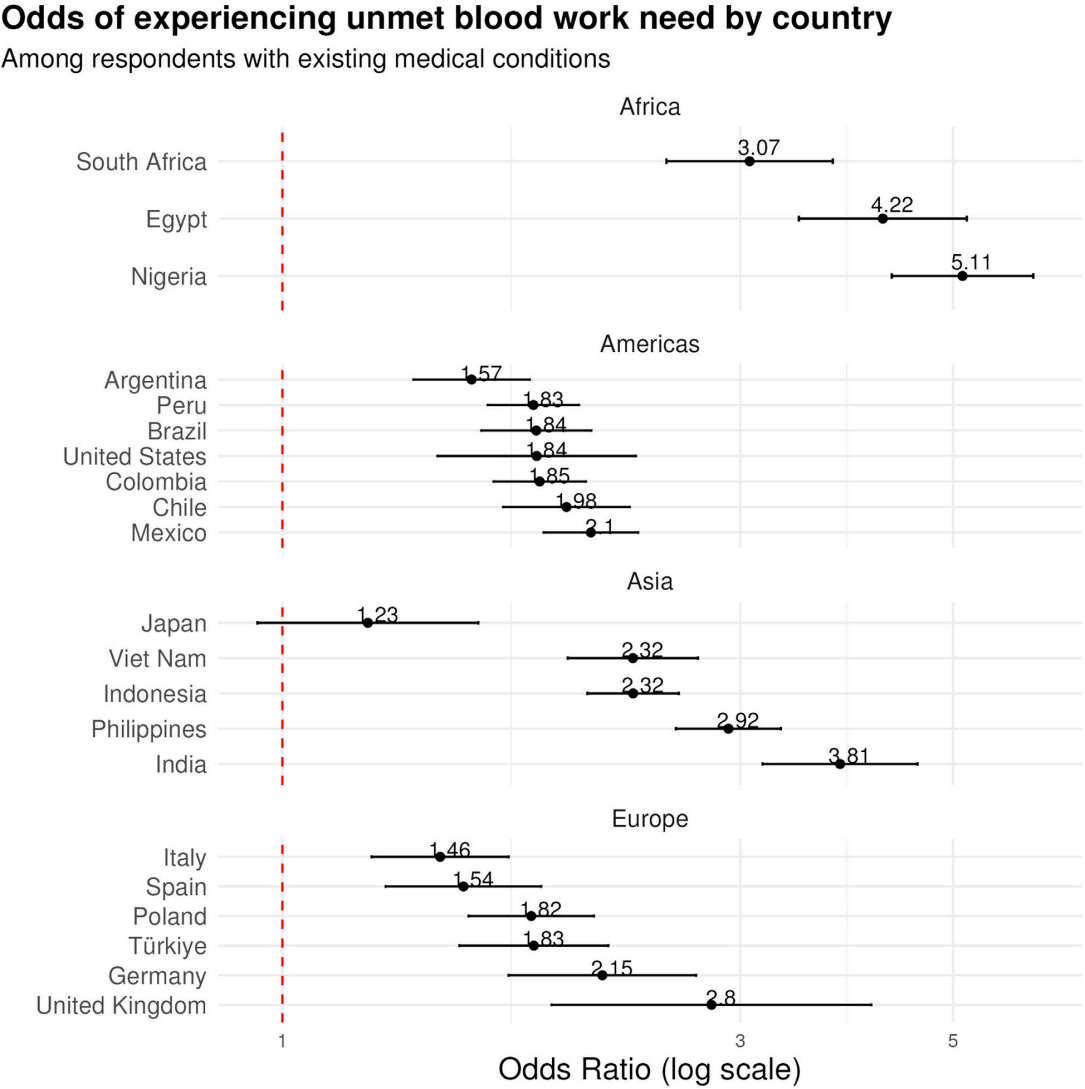
Odds ratios of unmet blood work need from 21 countries. This forest plot shows the results from a multivariable analysis of medical condition as a predictor of unmet blood work need. Values represent the odds ratios for unmet blood work need among respondents who have any medical condition, adjusted for demographic variables including gender, age, education, and financial status. Data are from the Pandemic Recovery Survey across 21 countries, 2023.

In our multivariable analysis, we analyzed data across all countries to evaluate the odds of unmet blood work needs among respondents with medical conditions, with adjustments made for demographic variables such as gender, age, education, and financial status. The findings revealed substantial variations across countries as shown in Figure 2.

In the Americas, Argentina had the lowest odds ratio (OR) in the region at 1.574 (95% CI: 1.370–1.810). Brazil, Peru, and Colombia exhibited similar levels of access challenges, with ORs of 1.839 (95% CI: 1.612–2.098), 1.826 (95% CI: 1.637–2.037), and 1.854 (95% CI: 1.660–2.071), respectively. Chile and Mexico demonstrated even higher ORs of 1.977 (95% CI: 1.698–2.301) and 2.096 (95% CI: 1.872–2.348).

In the South Asian region, Indonesia had an OR of 2.320 (95% CI: 2.079–2.589), while the Philippines showed a significantly higher OR of 2.916 (95% CI: 2.571–3.307). India presented an OR of 3.811 (95% CI: 3.165–4.590) and Vietnam had an OR of 2.318 (95% CI: 1.983–2.711).

European countries displayed varied impacts, with Italy having the lowest OR of 1.460 (95% CI: 1.239–1.721), Turkiye showed an OR of 1.828 (95% CI: 1.529–2.185), and Germany and the United Kingdom showed the highest ORs of 2.155 (95% CI: 1.721–2.698) and 2.799 (95% CI: 1.908–4.108).

In Africa, Nigeria had the highest observed OR of any country in the analysis at 5.113 (95% CI: 4.315–6.059). South Africa also showed high odds at 3.068 (95% CI: 2.513–3.746) and Egypt also exhibited a high OR of 4.223 (95% CI: 3.452–5.166).

The only country without statistical significance of unmet need between those with or without medical conditions, Japan, presented a relatively lower OR of 1.228 (95% CI: 0.942–1.600).

The high likelihood of delays in blood work for respondents with current medical conditions is an indicator for delays in diagnosis through blood testing. The detailed results below explore this indication further, comparing the proportion of respondents never receiving blood work by disease burden.

### Global Distribution of Medical Conditions That Require Blood Work for Diagnosis

DALYs of thalassaemias, sickle cell disorders, HIV, and malaria, and the SEVs of high fasting plasma glucose, impaired kidney function, and high LDL cholesterol are collected from the Global Burden of Disease 2021 framework [18].

DALYs of thalassemias (A), sickle cell disorders (B), HIV (E) and malaria (D) and SEVs of high fasting plasma glucose (F), impaired kidney function (G), and high LDL cholesterol (C) are shown in the distribution maps and tables in the Supplementary Appendix.

Using the Pearson correlation coefficient, correlation between the proportion of respondents never receiving blood work and the corresponding disease burden varies by disease, as shown in Table 2. High LDL cholesterol and HIV both have significant correlations with 95% certainty, and thalassaemias, sickle cell disorders, and malaria have significant correlations with 90% certainty.

**TABLE 2:** Correlation between the proportion of respondents never receiving blood work and each disease burden value. Data are from the Pandemic Recovery Survey, 21 countries, 2023.

Disease	Pearson correlation	p-value
LDL	−0.57	0.006***
Thal	0.39	0.083**
SCD	0.40	0.071**
Malaria	0.39	0.081**
HIV	0.43	0.050***
FPG	−0.10	0.665
Kidney	0.27	0.237

Table 2 shows the correlation and p-value between the proportion of respondents indicating that they have never received blood work and the disease burden across seven diseases. Disease burden is calculated as disability-adjusted life years (DALYs) rates (per 100,000), both sexes combined for thalassaemias, sickle cell disorders, malaria, and HIV, and age-standardised summary exposure values (SEV) rates (per 100,000), both sexes combined for high LDL cholesterol, high fasting plasma glucose, and impaired kidney function.

## Discussion

The findings from our analysis highlight major disparities in accessing timely blood work across 21 countries, with profound implications for the early detection and management of diseases that necessitate blood testing for diagnosis.

### Blood Work Availability and Disease Burden

Our study underscores a critical gap in the accessibility of blood work, particularly in countries like Egypt, Nigeria, India, and the Philippines, where over 60% of respondents reported never undergoing any form of blood testing. This gap is not only a reflection of healthcare system inefficiencies, but also points to broader socioeconomic and policy-related challenges. For instance, 77.7% of respondents in Nigeria indicated they have never received blood work, which is concerning given the high burden of malaria, points to systemic issues in healthcare delivery and access, exacerbated by economic constraints and infrastructural deficits [23].

In Egypt, the staggering rate of respondents who have never had blood work done (80%) signals an urgent need for healthcare policy reform and enhanced public health initiatives, further amplified by the country’s high burden of diseases requiring timely blood testing.

### Healthcare Policies and Interventions

The disparities in accessing blood work call for targeted healthcare policies and interventions aimed at improving access to blood work. Nigeria’s development of a National Essential Diagnostics List (NEDL) represents a step towards ensuring Nigerians have access to high quality and affordable diagnostic services [24].

India’s approach to establishing point-of-care blood test stations is a commendable idea to boosting access to referrals, testing, and treatment [25]. However, in action, staffing and equipment shortages lead to delays and cultural barriers and educational limitations remain a significant challenge.

### Barriers Against Blood Work in High-Burden Settings

Our study’s findings point to the need for policy interventions that address the factors impeding access to blood work. Strategies may include improving healthcare affordability, enhancing education around the importance of regular health checkups, and ensuring that healthcare services are designed to be inclusive and accessible to all. Another need for health system improvements was to develop databases for susceptible individuals, such as those with high fasting plasma glucose and high levels of LDL cholesterol, to have timely blood work. This cannot be done without popularising blood tests in routine and preventive medical screenings. By identifying barriers to blood work access, policymakers can devise better plans to reduce the amount of underdiagnosis. This is especially important when gauging health system expenses against particular diseases, where underdiagnosis can contribute to sudden overspending or misallocation of resources.

Other countries with high rates of unmet blood work needs include South Africa, Egypt, and the Philippines, all of which have existing policies encouraging blood donation, but limited guidelines over timely blood testing. Our study hopes to raise concerns over potential underdiagnosis of medical conditions due to delayed blood work and helps identify susceptible populations in communities to facilitate the implementation of routine blood testing.

### Limitations

The lack of accessible data about the timeliness of diagnosis for the seven medical conditions (thalassaemia, sickle cell disorders, malaria, HIV, high fasting plasma glucose, high LDL cholesterol, and impaired kidney function) poses a significant challenge to monitoring and addressing these delays. The absence of readily available data on the level of severity of the seven types of medical conditions at the time of diagnosis hampers efforts to understand and improve the timeliness of blood work, especially in regions with the highest DALY rates for blood diseases. We included analysis by DALY rates of four types of diseases, thalassaemias, sickle cell disorders, malaria, and HIV, and SEV rates of three types of conditions – high fasting plasma glucose, high LDL cholesterol, and impaired kidney function – which require blood tests for primary diagnosis and monitoring. High rates of DALYs and SEVs faced by countries such as Egypt, Nigeria, and India, where over 70% of respondents have never received blood work, underscores the importance of establishing protocols for timely blood tests to reduce potential underdiagnosis.

One strength of our study is the wide scope of geographical coverage, where 21 countries are included. We analysed survey results surrounding the socioeconomic information of respondents and delays in blood work they may or may not have experienced. The level of detail helps uncover the characteristics of populations experiencing delays in blood work, as well as identify the countries with high rates of unmet blood work needs.

While the data from the Pandemic Recovery Survey are self-reported by nature, there has been good convergence of self-reports and reliable administrative medical records [26] to warrant such comparison.

### Conclusion

Our study underscores the urgent need to address disparities in access to timely blood work. By understanding the socioeconomic factors that contribute to these delays, and by improving the availability of health data, we can help prioritise equitable access to diagnostic services to reduce the burden of blood diseases and improve health outcomes for all populations.

These discussions should pave the way for future research and policy discussions aimed at bridging the gaps in healthcare access revealed by our study.

## Data Availability

Deidentified participant-level microdata are available for download on GESIS: https://doi.org/10.7802/2631.
