# Supplementary material for: Delays in Blood Work and Disease Burden: A Cross-Sectional Analysis of Unmet Blood Work Need and Seven Key Health Conditions Across 21 Countries
Source: Int J Public Health. 2025 Jan 6;69:1607667. doi: 10.3389/ijph.2024.1607667 (PMC11742933; doi:10.3389/ijph.2024.1607667)
Supplement: Supplementary file 1 [file DataSheet1.docx]

Appendix

**Age-standardised DALY rates (per 100 000) by location, both sexes, combined, 2021 for A. thalassaemias, B. sickle cell disorders, D. malaria, E. HIV**

**Age-standardised SEV rates (per 100,000) by location, both sexes combined, 2021 for C. high LDL cholesterol, F. high fasting plasma glucose, G. impaired kidney function**


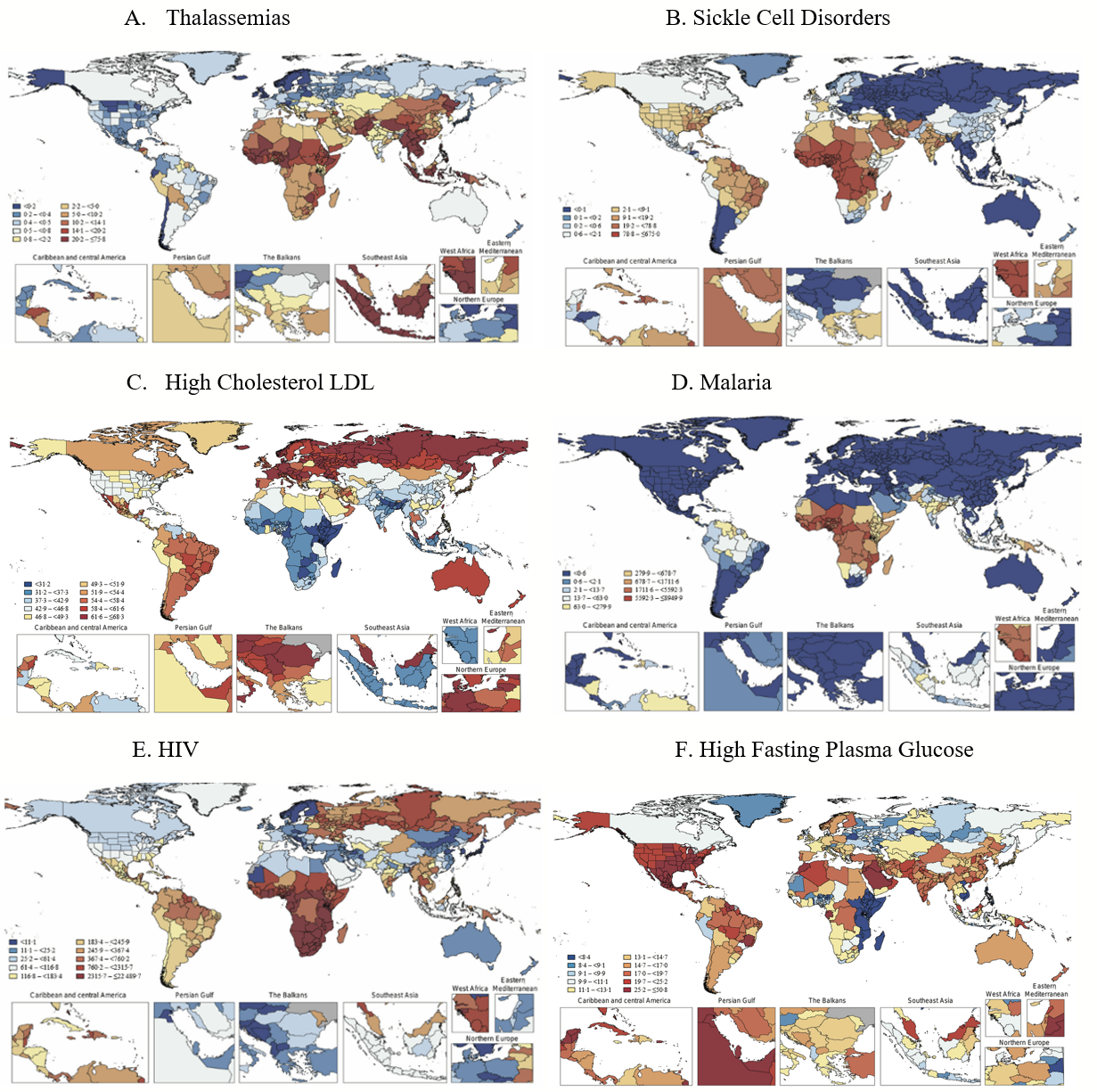


**
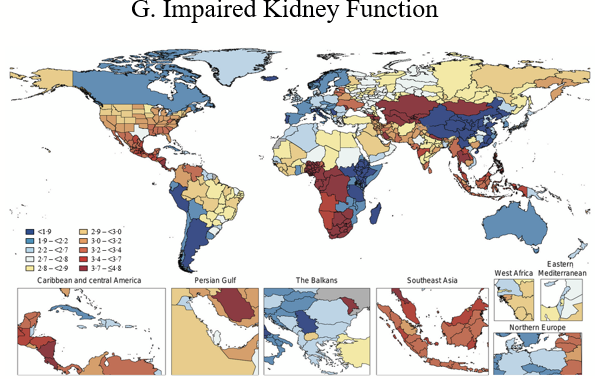
**

**DALY rates (per 100 000) for thalassaemias, sickle cell disorders, malaria, and HIV, and SEV rates for high LDL cholesterol, high fasting plasma glucose, impaired kidney function.**

| Country name | Thalassemia | Sickle Cell Disorder | Malaria | HIV | High Fasting Plasma Glucose | Impaired Kidney Function | High LDL |
| --- | --- | --- | --- | --- | --- | --- | --- |
| Argentina | 0.00001 | 0.00000 | 0.00000 | 0.00193 | 0.111 | 0.191 | 0.41515 |
| Brazil | 0.00001 | 0.00016 | 0.00006 | 0.00304 | 0.115 | 0.212 | 0.45121 |
| Chile | 0.00000 | 0.00000 | 0.00000 | 0.00131 | 0.137 | 0.204 | 0.39790 |
| Colombia | 0.00000 | 0.00006 | 0.00011 | 0.00282 | 0.151 | 0.287 | 0.39474 |
| Egypt | 0.00005 | 0.00015 | 0.00000 | 0.00024 | 0.136 | 0.283 | 0.33762 |
| Germany | 0.00000 | 0.00001 | 0.00000 | 0.00025 | 0.157 | 0.161 | 0.47906 |
| India | 0.00001 | 0.00014 | 0.00091 | 0.00136 | 0.151 | 0.236 | 0.24118 |
| Indonesia | 0.00028 | 0.00000 | 0.00028 | 0.00068 | 0.091 | 0.271 | 0.23669 |
| Italy | 0.00003 | 0.00001 | 0.00000 | 0.00045 | 0.130 | 0.157 | 0.44009 |
| Japan | 0.00000 | 0.00000 | 0.00000 | 0.00007 | 0.076 | 0.214 | 0.41222 |
| Mexico | 0.00000 | 0.00001 | 0.00000 | 0.00213 | 0.208 | 0.372 | 0.41263 |
| Nigeria | 0.00018 | 0.00338 | 0.06007 | 0.02319 | 0.063 | 0.182 | 0.25793 |
| Peru | 0.00004 | 0.00002 | 0.00005 | 0.00219 | 0.077 | 0.202 | 0.35069 |
| Philippines | 0.00013 | 0.00000 | 0.00001 | 0.00216 | 0.093 | 0.300 | 0.37083 |
| Poland | 0.00000 | 0.00000 | 0.00000 | 0.00017 | 0.132 | 0.197 | 0.39196 |
| South  Africa | 0.00008 | 0.00000 | 0.00007 | 0.09365 | 0.132 | 0.226 | 0.27355 |
| Spain | 0.00001 | 0.00001 | 0.00000 | 0.00064 | 0.132 | 0.143 | 0.40214 |
| Türkiye | 0.00008 | 0.00005 | 0.00000 | 0.00016 | 0.113 | 0.260 | 0.30983 |
| United  Kingdom | 0.00000 | 0.00002 | 0.00000 | 0.00028 | 0.183 | 0.148 | 0.43509 |
| United  States | 0.00000 | 0.00009 | 0.00000 | 0.00095 | 0.166 | 0.216 | 0.31264 |
| Viet  Nam | 0.00021 | 0.00000 | 0.00000 | 0.00251 | 0.102 | 0.259 | 0.29130 |

**All rates are age-standardised (per 100 000) by location, both sexes, combined, from the Global Burden of Disease Study, 2021.**
